# Supplementary figures and images for: The Development of Ovine Gastric and Intestinal Organoids for Studying Ruminant Host-Pathogen Interactions
Source: Front Cell Infect Microbiol. 2021 Sep 8;11:733811. doi: 10.3389/fcimb.2021.733811 (PMC8456012; doi:10.3389/fcimb.2021.733811)

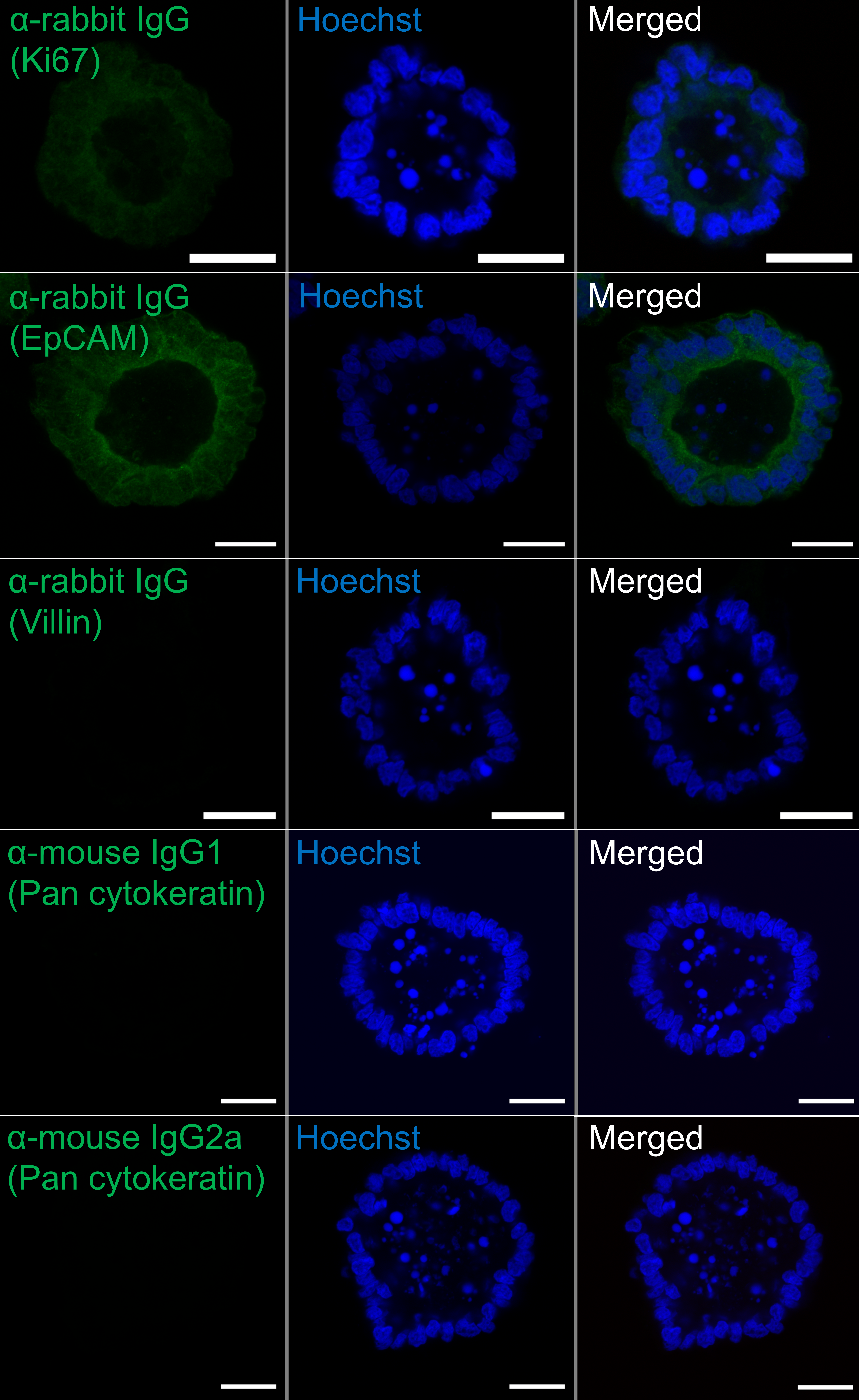

Supplement: Supplementary Figure 1 — Negative controls for immunofluorescence antibody labelling in abomasum organoids. Representative confocal microscopy images of abomasum organoids probed with non-specific host IgG followed by indirect Alexa Fluor® 488-conjugated secondary antibody labelling. Marker name in green brackets indicates the antibody labelling control each organoid image represents. Scale bars = 10 µm. [file Image_1.tif]

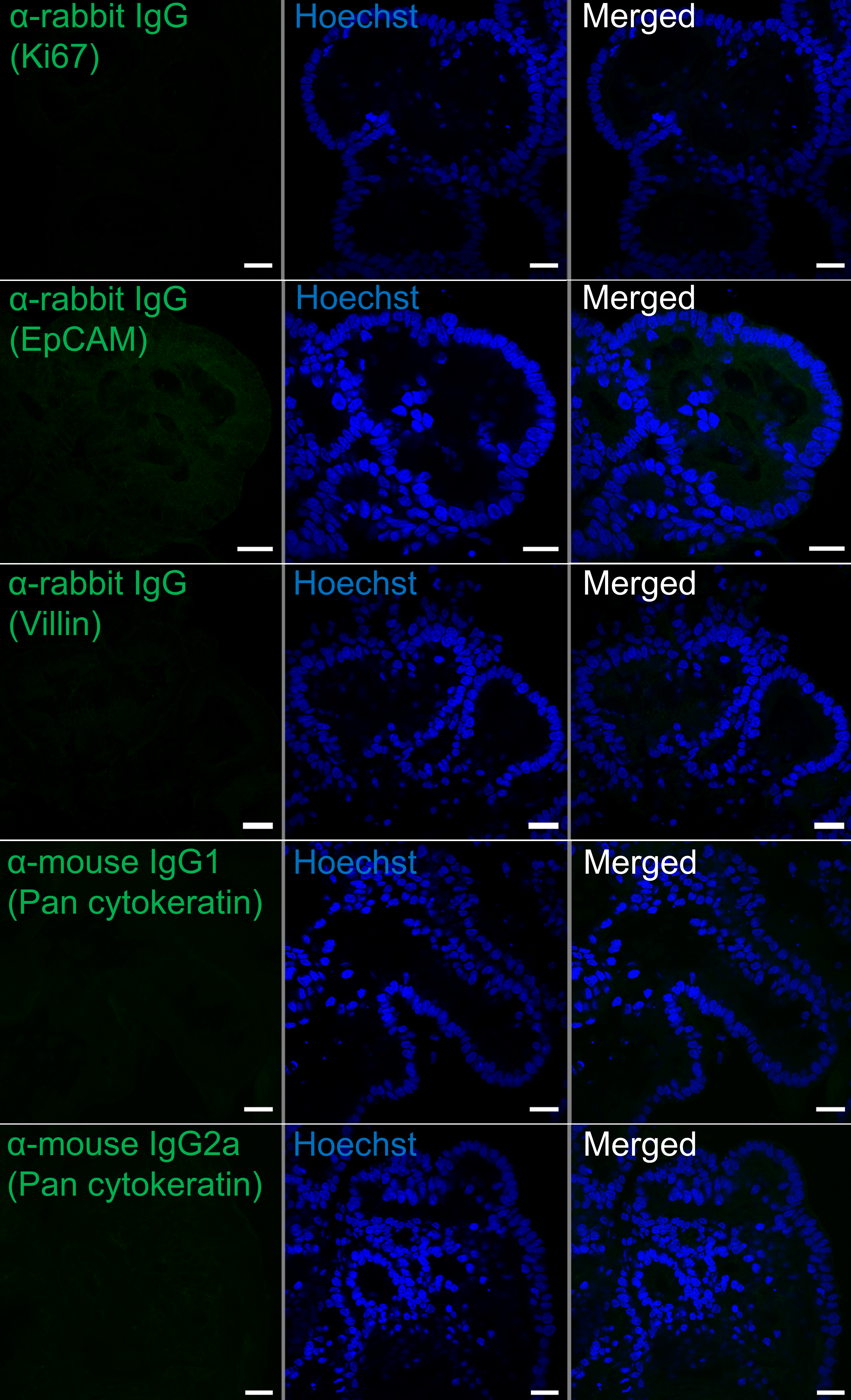

Supplement: Supplementary Figure 2 — Negative controls for immunofluorescence antibody labelling in intestinal organoids. Representative confocal microscopy images of ileum organoids probed with non-specific host IgG followed by indirect Alexa Fluor® 488-conjugated secondary antibody labelling. Marker name in green brackets indicates the antibody labelling control each organoid image represents. Hoescht, blue. Scale bars = 10 µm. [file Image_2.tif]

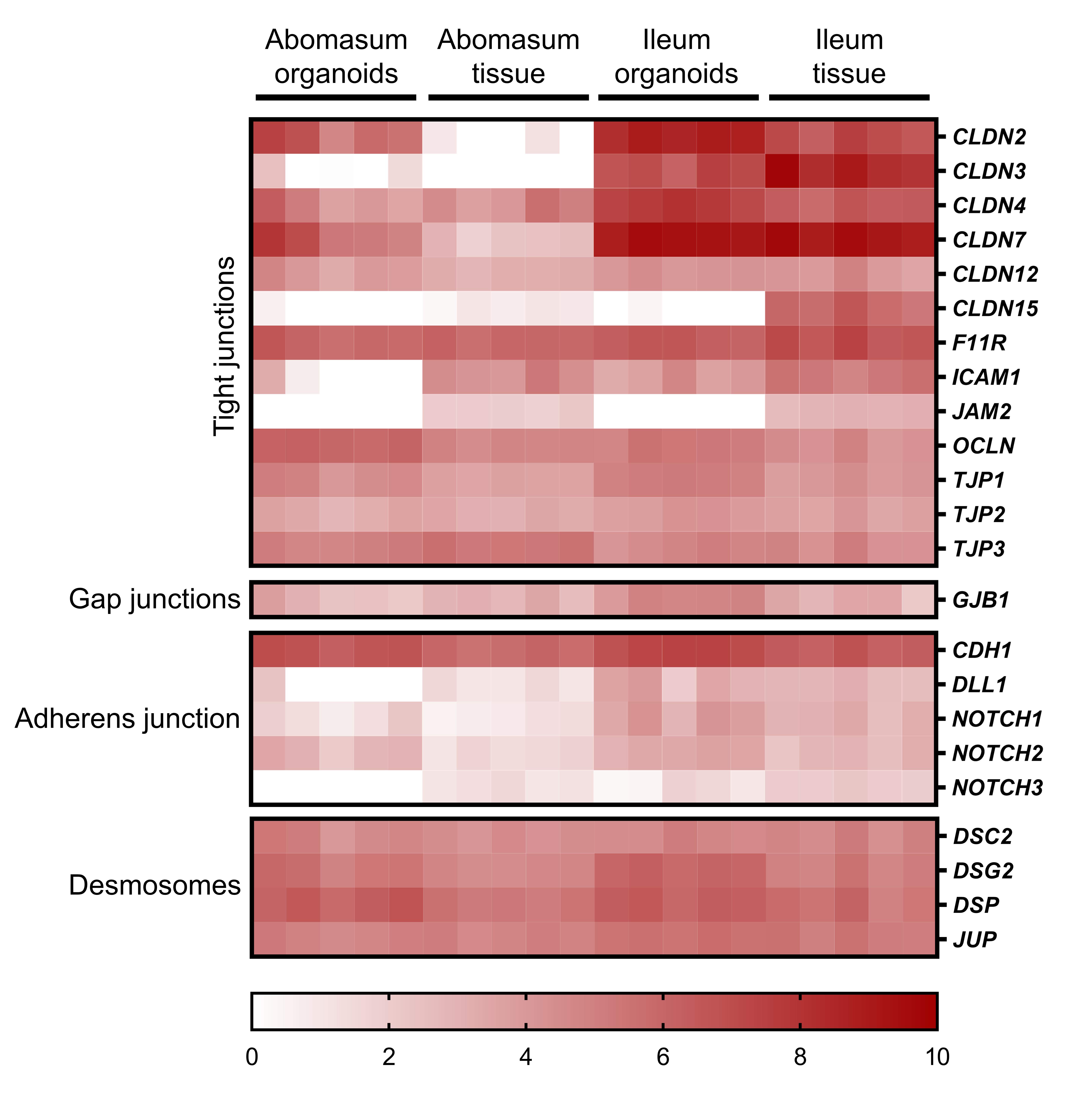

Supplement: Supplementary Figure 3 — Heat map showing the expression of cell junction-related genes in abomasum and ileum tissue and organoids. RNA-seq analysis was performed to compare gene expression in abomasal and ileal tissue derived from five lambs and abomasum and ileum organoids across multiple passages. Squares from left to right under “abomasum tissue” and “ileum tissue” represent lambs T1-T5. Squares from left to right under abomasum organoids and ileum organoids represent passages P0-P4. Scale = log2 transcripts per million reads. Details of genes included in the heat map, including ENSOART sequence identifiers, are shown in Supplemental File 2. [file Image_3.tif]

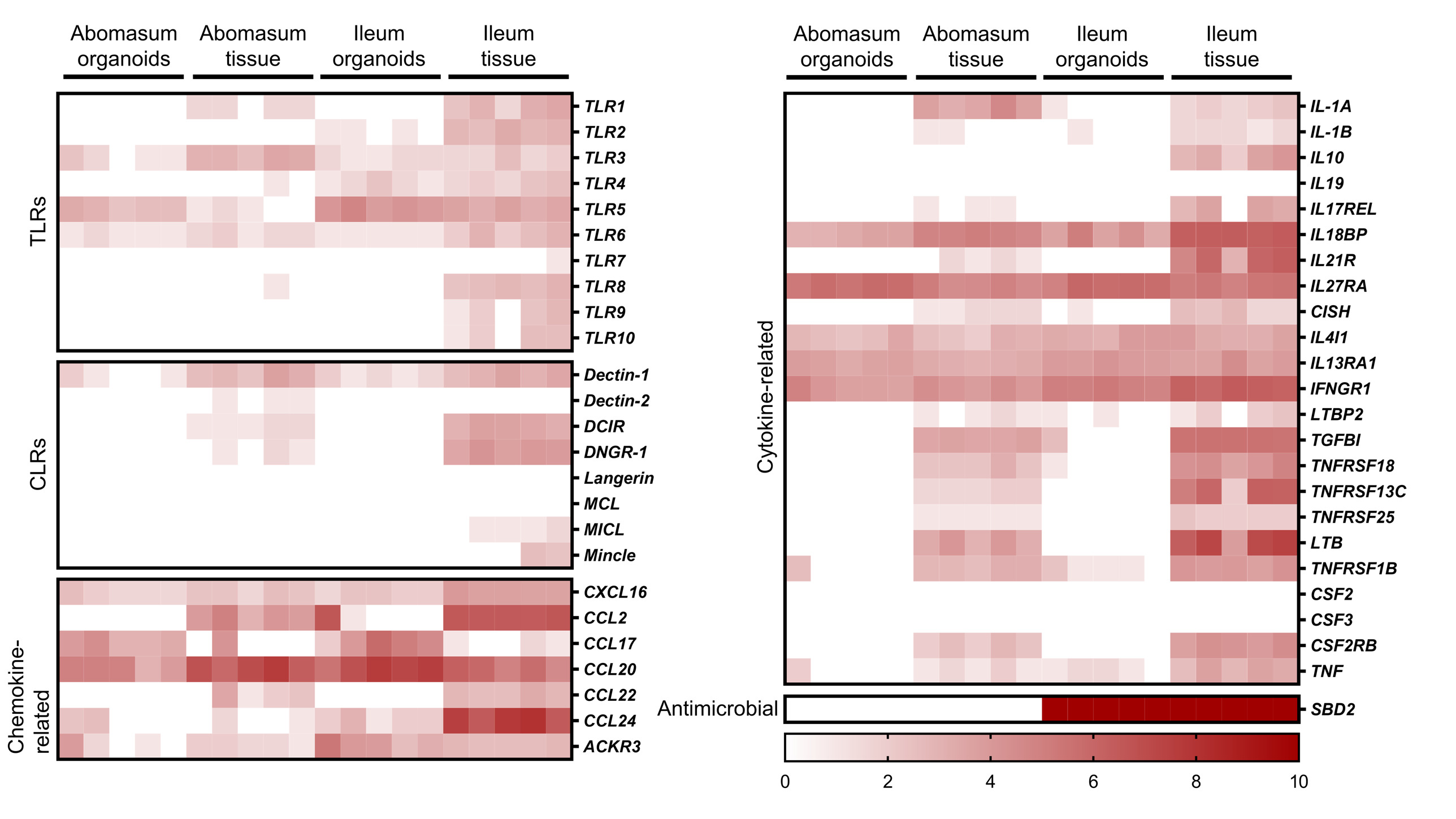

Supplement: Supplementary Figure 4 — Heat map showing the detection of immune-related gene expression in abomasum and ileum tissue and organoids. RNA-seq analysis was performed to compare gene expression in abomasal and ileal tissue derived from five lambs and abomasum and ileum organoids across multiple passages. Squares from left to right under “abomasum tissue” and “ileum tissue” represent lambs T1-T5. Squares from left to right under abomasum organoids and ileum organoids represent passages P0-P4. Scale = log2 transcripts per million reads. TLRs, toll-like receptors; CLRs, C-type lectin receptors. Details of genes included in the heat map, including ENSOART sequence identifiers, are shown in Supplemental File 2. [file Image_4.tif]
